# Hypomagnesemia predicts postoperative biochemical hypocalcemia after thyroidectomy

**DOI:** 10.1186/s12893-017-0258-2

**Published:** 2017-05-25

**Authors:** Han Luo, Hongliu Yang, Wanjun Zhao, Tao Wei, Anping Su, Bin Wang, Jingqiang Zhu

**Affiliations:** 1 Department of Thyroid & Parathyroid surgery, West China Hospital, Sichuan University, Guoxue Alley 37#, Chengdu, Sichuan 610041 People’s Republic of China; 2 Department of Nephrology, West China Hospital, Sichuan University, Chengdu, People’s Republic of China; 3 Biostatistics Center, West China Hospital, Sichuan University, Chengdu, People’s Republic of China

**Keywords:** Hypomagnesemia, Relative decline of iPTH, Hypocalcemia, Symptom, Female

## Abstract

**Background:**

To investigate the role of magnesium in biochemical and symptomatic hypocalcemia, a retrospective study was conducted.

**Methods:**

Less-than-total thyroidectomy patients were excluded from the final analysis. Identified the risk factors of biochemical and symptomatic hypocalcemia, and investigated the correlation by logistic regression and correlation test respectively.

**Results:**

A total of 304 patients were included in the final analysis. General incidence of hypomagnesemia was 23.36%. Logistic regression showed that gender (female) (OR = 2.238, *p* = 0.015) and postoperative hypomagnesemia (OR = 2.010, *p* = 0.017) were independent risk factors for biochemical hypocalcemia. Both Pearson and partial correlation tests indicated there was indeed significant relation between calcium and magnesium. However, relative decreasing of iPTH (>70%) (6.691, *p* < 0.001) and hypocalcemia (2.222, *p* = 0.046) were identified as risk factors of symptomatic hypocalcemia. The difference remained significant even in normoparathyroidism patients.

**Conclusions:**

Postoperative hypomagnesemia was independent risk factor of biochemical hypocalcemia. Relative decline of iPTH was predominating in predicting symptomatic hypocalcemia.

## Background

Postoperative hypocalcemia is a well-recognized complication after thyroidectomy, of which reportedly, prevalence ranges from 10% to 83% [1–7]. And symptomatic hypocalcemia (SxH) occurs in 10% to 36% [2, 4], including spasm, muscular cramp, Chevok’s syndrome. Multiple factors, including surgery technique, different definitions of hypocalcemia, and calcium or vitamin D supplement et al. contribute to the wide variation. It is known that risk factors of hypocalcemia have been investigated extensively in recent years. However, the role of hypomagnesemia in hypocalcemia is revealed limitedly.

Meanwhile, magnesium is a crucial element in human metabolism pathway, acting as cofactor in enzymatic reaction [8]. Reportedly, magnesium could affect calcium level via modulating PTH secretion and PTH receptor sensitivity, as well as calcium excretion in kidney [9, 10]. Post-thyroidectomy hypomagnesemia has been approved to reduce the production of PTH, decrease the affinity of PTH receptor and production of vitamin D, all of which may lead to hypocalcemia [10]. So we guess magnesium will affect calcium level after thyroidectomy. Previously, a few researchers have investigated the association between magnesium and calcium level and hypothyroidism after thyroidectomy [3, 4]. But all of them had small recruitment in retro/prospective study. Moreover, literature about the effect of hypomagnesemia is limited. Therefore, value of magnesium in prediction and treatment of hypocalcemia was underestimated now.

Therefore, we expect to explore the relationship between hypomagnesemia and biochemical hypocalcemia and symptomatic hypocalcemia in the present study.

## Methods

The retrospective study was conducted based on the thyroid surgery department database, which was maintained prospectively.

Patients were recruited between June 2014 and July 2015. Patients who underwent thyroidectomy (total [TTx] and completion thyroidectomy [CT]) were included in the retrospective study. And preoperative and postoperative data about calcium, magnesium and PTH was extracted from the database. Exclusion criteria were lobectomy and near TTx, or any disease which would affect the level of calcium and magnesium, or patients with preoperative hyper/hypo-calcemia/magnesemia.

In order to make sure the baseline is comparative as much as possible, we only extracted records from Dr. JQZ, one of experienced surgeon with high volume of more than 600 cases annually. Conventional capsular dissection technique was adopted guided by principle “1 + X” treating parathyroid, which means at least one parathyroid gland was readily recognized and preserved in situ, and not sought out systematically. Any parathyroid attached on the dorsal side of thyroid were freed carefully and retained in situ with intact vasculature. If parathyroid gland is discolored at the end of surgery, autotransplantation gland will be performed.

A complete central nodal dissection (CND) was performed firstly on the side of carcinoma. Supposed that intraoperative frozen section confirmed more than 1 positive lymph node (ipsilateral paratracheal, pretracheal and prelaryngeal nodes) in the central compartment, a contralateral CND would follow. We did bilateral CND for bilateral foci or isthmus foci. Patients with lateral neck lymph nodes metastases will undergo concurrent unilateral or bilateral modified lateral neck dissection on the basis of preoperative FNA study, compartment of the central neck is bounded superiorly by the thyroid cartilage, inferiorly by the innominate artery, medially by the trachea, and laterally by the carotid artery (level VI-VII) [11].

Blood was collected and tested in 6 AM in the 1st postoperative morning---postoperative calcium, magnesium and iPTH was recorded. Then normal calcemic patients without any discomfort were discharged in 1st or 2nd postoperative day. The other patients were prescribed with oral calcium and/or vitamin D supplementation on case-by-case basis. Generally, whether supply with calcium and/or vitamin D was decided by the hypocalcemic symptom or biochemical hypocalcemia.

Averagely, each patient received 500 mL total intravenous fluid replacement after surgery until he (she) could drink. Tumor staging system adopted standard of Union for International Cancer Control (UICC) sixth edition. Relative decrease of iPTH = (preoperative iPTH-postoeprative iPTH)/preoperative iPTH. Hypomagnesemia, hypocalcemia means below the reference, reference: magnesium 0.67–1.04 mmol/L, calcium 2.1–2.7 mmol/L and iPTH 1.6–6.9 pmol/L. SxH means specific numbness, spasm, muscular cramp and Chevok’s syndrome et al. led by hypocalcemia.

Patients were followed up rigorously at outpatient department, and begin from the end of 1st month after discharge. In terms of patients administrated with calcium and/or vitamin D were followed every month after discharge until 6 months, then 6 months thereafter. Or they were followed 1, 3, 6 month(s) after discharge until 6 months, then 6 months thereafter.

Data analysis was performed by SPSS version 21 (SPSS Inc., Chicago, IL). If normal distributed, continuous variables were presented as the mean ± standard deviation and compared by t-test; if not, variables were presented as median (interquartile range) and compared by Mann-Whitney U-test. Pearson Chi-square test or Fisher’s exact test was used to compare frequency (percentage) for categorical variables. Logistic regression was used to determine the risk factor. *P* value <0.05 indicated significant difference.

The ethics approve was waived by the Ethic Committee of West China Hospital, Sichuan University (Chengdu, China). The informed consent forms were obtained from all individual participants included in this study. All study participants provided written informed consent to indicate their agreement for the clinical data to be used in clinical research and publication.

## Results

A total of 304 patients (85 male vs 219 female), with mean age 41.6, were enrolled in the final analysis basing on the inclusion and exclusion criteria. 298 (about 98%) patients were diagnosed with malignant thyroid tumor (papillary, medullary and follicular thyroid cancer). Nearly all of patients underwent TTx in the present study (301/304, 99.01%) and only 3 patients underwent CT. Autotransplantation of parathyroid gland (PTG) was performed in 179 patients with 188 glands. 195, 105 and 47 patients underwent bilateral CND, unilateral CND and lateral nodal dissection (LND) respectively, Table 1. No patient had hypomagnesemia preoperatively, while 71 (23.36%) patients developed hypomagnesemia post-thyroidectomy. And given of just 3 cases emergency calcium infusion after charge during follow-up, it is hard to perform multivariate analysis about emergency infusion. We only analyzed the risk factor of biochemical and symptomatic hypocalcemia.

In terms of biochemical hypocalcemia, patients were divided into two groups based on the presence and absence of biochemical hypocalcemia (82 vs 222), Table 2. General incidence of biochemical hypocalcemia was 26.97%. Gender (female) [2.284 (1.203, 4.334)] and postoperative (Po) hypomagnesemia [2.158 (1.227, 3.797)] was identified as risk factor through univariate analysis. Multivariate analysis showed gender (female) [OR = 2.238, 95% CI (1.171, 4.276), *p* = 0.015] and Po hypomagnesemia [OR = 2.010, 95% CI (1.131, 3.570), *p* = 0.017] were identified as independent risk factors for biochemical hypocalcemia, Table 3. Incidence of hypomagnesemia in female and male patients was 26.03% and 16.47% respectively.

**Table 1:** Basic characteristics of patients enrolled

Age	41.62±11.50
Sex (male/female)	85/219
Preoperative magnesium	0.88±0.11
Postoperative magnesium	0.73±0.076
Preoperative calcium	2.21±0.13
Postoperative calcium	2.16±0.17
Thyroidectomy (TTx/CT)	301/3
Autotransplantation of PTG	179/125
Identification of PTG (≥3/<3)	274/30
Pathological diagnosis (malignancy/benign)	298/6
Extent of CND(bilateral/unilateral/non)	195/105/4
Lateral nodal dissection	47/257
Preoperative iPTH	5.48±2.06
Postoperative iPTH	2.24±1.44

*TTx* total thyroidectomy, *CT* completion thyroidectomy, *PTG* parathyroid gland, *CND* central nodal dissection, *iPTH* intact parathyroid hormone

**Table 2:** Baseline comparison between biochemical hypocalcemia and eucalcemia patients

	Hypocalcemia (82)	Normocalcemia (222)	*P* value
Age (>45/<45)	32/50	71/151	0.276
Sex(male/female)	14/68	71/151	0.01
Po hypomagnesemia	28/54	43/179	0.009
Po hyperphosphatemia	41/41	89/133	0.151
Pathological diagnosis(malignancy/benign)	82/0	216/6	0.196
TTx/CT	81/1	220/2	>0.999
bilateral/unilateral CND	53/29	142/76	>0.999
TTx+BCND	52/30	137/85	0.791
Lateral nodal dissection	11/71	36/186	0.597
Autotransplantataon of PTG	52/30	127/95	0.36
Identification of PTG (≥3/<3)	65/17	209/13	>0.999
T1-T2/T3-T4	36/46	108/108	0.366
Po iPTH<1.6 pmol/L	36/46	71/151	0.059
Relative decline of iPTH>70%	35/47	73/149	0.137
Thyroiditis	13/69	28/194	0.455
Hyperthyroidism	2/80	6/216	>0.999

*TTx* total thyroidectomy, *CT* completion thyroidectomy, *Po* postoperative, *PTG* parathyroid gland, *CND* central nodal dissection, *BCND* bilateral central nodal dissection, *iPTH* intact parathyroid hormone

**Table 3:** Risk factor identification of biochemical hypocalcemia

	Univariate		Multivariate		
	OR	*P* value	OR	95% CI	*P* value
Age (>45/<45)	1.361	0.250			
Sex(male/female)	0.438	0.012	2.150	1.126, 4.104	0.020
Po hypomagnesemia	2.158	0.008	2.030	1.146, 3.595	0.015
Po hyperphosphatemia	1.494	0.122			
Pathological diagnosis (malignancy/benign)	/	>0.999			
TTx/CT	0.736	0.804			
bilateral/unilateral CND	/	>0.999			
TTx + BCND	1.075	0.786			
Lateral nodal dissection	0.800	0.549			
T1-T2/T3-T4	0.783	0.347			
Po iPTH < 1.6 pmol/L	1.664	0.055			
Relative decline of iPTH > 70%	1.520	0.114			
Thyroiditis	0.766	0.464			
Hyperthyroidism	1.111	0.899			
Autotransplantataon of PTG	1.297	0.330			
Identification of PTG (≥3/<3)	1.342	0.765			

*TTx* total thyroidectomy, *CT* completion thyroidectomy, *Po* postoperative, *PTG* parathyroid gland, *CND* central nodal dissection, *BCND* bilateral central nodal dissection, *iPTH* intact parathyroid hormone, *OR* odds ratio, *CI* Confidence Interval

**Table 4:** Risk factor identification of symptomatic hypocalcemia

	Univariate		Multivariate		
	OR	*P* value	OR	95% CI	*P* value
Age (>45/<45)	0.771	0.493			
Sex(male/female)	1.837	0.166			
Po hypocalcemia	2.446	0.014	2.222	1.013, 4.877	0.046
Po hyperphosphatemia	1.237	0.540			
Po hypomagnesemia	2.689	0.047	1.376	0.973, 2.011	0.218
Pathological diagnosis (malignancy/benign)	3.639	0.144			
TTx/CT	3.568	0.304			
bilateral/unilateral CND	1.432	0.344			
TTx+BCND	1.369	0.397			
Lateral nodal dissection	1.276	0.590			
T1-T2/T3-T4	0.679	0.275			
Po iPTH<1.6 pmol/L	4.949	<0.001	1.194	0.897, 1.901	0.128
Relative decline of iPTH>70%	6.510	<0.001	6.691	3.033, 14.759	<0.001
Thyroiditis	0.649	0.344			
Hyperthyroidism	1.000	>0.999			
Autotransplantataon of PTG	0.625	0.204			
Identification of PTG (≥3/<3)	0.986	0.121			

*TTx* total thyroidectomy, *CT* completion thyroidectomy, *Po* postoperative, *PTG* parathyroid gland, *CND* central nodal dissection, *BCND* bilateral central nodal dissection, *iPTH* intact parathyroid hormone, *OR* odds ratio, *CI* Confidence Interval

**Table 5:** Comparison in incidence of SxH in normoparathyroidism patients

	Po iPTH≥1.6 pmol/L *N*=197			Po iPTH≥1 pmol/L *N*=237		
	<70% *N*=176	>70% *N*=21	*P* value	<70% *N*=194	>70% *N*=43	*P* value
Age (>45)	57 (32.39%)	10 (47.62%)	>0.999	62 (31.96%)	16 (37.21%)	0.591
Sex(male)	63 (35.80%)	5 (23.81%)	0.337	66 (34.02%)	9 (20.93%)	0.106
Po hypomagnesemia	29 (16.48%)	5 (23.81%)	0.372	35 (18.04%)	12 (27.91%)	0.145
Po hypocalcemia	41 (23.30%)	5 (23.81%)	>0.999	46 (23.71%)	10 (23.26%)	>0.999
TTx	175 (99.43%)	20 (95.24%)	0.202	193 (99.48%)	42 (97.67%)	0.331
bilateral CND	113 (64.20%)	11 (52.38%)	0.341	129 (66.49%)	24 (55.81%)	0.365
SxH	10 (5.68%)	4 (19.05%)	0.047	10 (5.15%)	7 (16.28%)	0.019

*TTx* total thyroidectomy, *CT* completion thyroidectomy, *Po* postoperative, *CND* central nodal dissection, *iPTH* intact parathyroid hormone, *SxH* symptomatic hypocalcemia

The Pearson test indicated that there was a significant correlation between Po calcium and Po magnesium (*R* = 0.208, *p* < 0.001). Po iPTH level, while, in hypomagnesemic patients was significantly lower than normal magnesium patients in present study (1.75 ± 1.40 vs 2.39 ± 1.42, *p* = 0.001). Given of the complicate relation among iPTH, magnesium and calcium, partial correlation showed partial correlation coefficient between calcium and magnesium remained significant (*R* = 0.166, *p* = 0.004) when variable-iPTH was controlled.

Regarding to SxH, a total of 38 patients (12.5%) were identified as SxH after surgery. Hypomagnesemia [2.446, (1.197, 4.495)], hypocalcemia [2.689, (1.012, 7.144)], iPTH < 1.6 pmol/L [4.949, (2.380, 10.291)], and relative decrease of iPTH > 70% [6.510 (3.020, 14.034)] were identified as risk factors for SxH through univariate analysis. While, only relative decrease of iPTH > 70% [6.691, (3.033, 14.759), *p* < 0.001] and hypocalcemia [2.222, (1.013, 4.877), *p* = 0.046] remained significantly, Table 4. Besides, Pearson correlation analysis revealed significant correlation between decreasing percent of iPTH and SxH (*R* = 0.232, *p* < 0.001). 28 patients developed SxH in patients with relative decreasing >70% (25.93%, 28/108), by contrast, 10 patients developed SxH in patients <70% (5.10%, 10/196).

In order to furtherly investigate the role of relative decreasing of iPTH in SxH, patients with anomaly PO iPTH (<1.6 pmol/L) were excluded. No significant difference was found in age, gender and hypomagnesemia, however, relative decrease of SxH showed significant difference between two groups (10/166 vs 4/17, *p* = 0.047). Furthermore, when excluded the patients with iPTH < 1 pmol/L, incidence of SxH remained significantly different, *p* = 0.019, Table 5.

## Discussion

The purpose of the retrospective study was to investigate the risk factors of hypocalcemia. Though the topic was extensively studied, consensus was hardly to reach. The present study explored the risk factors of biochemical hypocalcemia, symptomatic hypocalcemia. Our findings would reveal that hypomagnesemia is indeed strongly associated with biochemical hypocalcemia, but not SxH, which has significant independent relation with relative decrease of iPTH.

Reportedly, prevalence of biochemical and symptomatic hypocalcemia was extensively various. This variability of prevalence is much associated with adopted definition of hypocalcemia, dissection technique, included extent of thyroidectomy and supplement plan of calcium and vitamin D post-thyroidectomy. It is believed that the overall rate of biochemical hypocalcemia (26.97%) [12–15] and SxH (12.50%) [4, 12, 14, 16] in our study matches well with previously published result-biochemical (21%–50%)/symptomatic (7%–36%).

As we know, the relation between calcium and magnesium metabolism is complicated. Magnesium deficiency is associated with impaired secretion and affinity of PTH. Magnesium may compete with calcium, and play a mimic effect on the parathyroid cell. The “calcium” receptor stimulates secretion of PTH in the presence of elevated level of calcium. However, when hypomagnesemia, calcemic ions are relative much more than usual, secretion of PTH is inhibited. Rude et al. suggested that intravenous injection of magnesium solution, when hypocalcemia secondary to hypomagnesemia, secretion of PTH would increase dramatically in 1 min after administration [10]. It approved conversely, hypomagnesemia could inhibit PTH secretion. Cherian et al. reported that lower magnesium level among patients with abnormal postoperative PTH than those with normal PTH in a series of 50 patients, yet it was not significant (*p* = 0.21) [17]. While, postoperative iPTH in eumagnesemia patients was lower than that in hypomagnesemia patients significantly in the present study (1.75 ± 1.40 vs 2.39 ± 1.42, *p* = 0.001).

Clinically, limited literature had investigated the role of hypomagnesemia after thyroidectomy. Wilson et al. reported that hypomagnesemia indeed had a significant relation with hypocalcemia in a prospective study in a series of 50 patients with total thyroidectomy [4]. And Hammerstad et al. suggested that decreasing degree of magnesium level in 48 h after operation may predict development of permanent hypoparathyroidism combined with preoperative serum calcium and postoperative PTH [3]. In our present study, we also found that the significant relation between hypomagnesemia and hypocalcemia (*p* = 0.017, OR = 2.010). Being different from the Cherian’s research, in which the prevalence of hypomagnesemia pre/post-thyroidectomy was about 24% and 70% respectively, only 23.36% patients developed hypomagnesemia after surgery, and no patient had hypomagnesemia [17]. The obvious statistic gap may attribute to the totally different environment and dietary habit.

In addition, gender (female) was proved to be an independent risk factor for hypocalcemia in the present study. Likely, Wilson et al. also reported that there was a trend to lower calcium levels in female patients (*p* = 0.039), though not positively related in multivariate analysis [4]. Different level of magnesium between genders may be the main reason. Syedmoradi et al. reported that hypomagnesemia was much prevalent in women (6.0%) than man (3.2%), *p* < 0.05 in cross-sectional study with 1558 subjects in Iranian urban area [18]. Moreover, 59% of Chinese lactating women intakes fewer magnesium than average requirement in a cross-sectional survey of three cities [19]. Therefore, we suspected that female were vulnerable to hypomagnesemia (OR = 2.238, *p* = 0.015) may result from high prevalence of hypomagnesemia in female patients (female vs male: 26.03% vs 16.47%). Sands concluded that Female gender as a risk factor for transient post-thyroidectomy hypocalcemia [20].

Regarding to SxH, relative decrease of iPTH (>70%) was predominate factor. Calan and Schlottmann. F also concluded that patients with less than 80% drop in iPTH levels can be safely discharged the day of surgery [21]. Besides, Puzziello et al. reported that more than 62% decreasing of iPTH in 2 h after surgery, though normocalcemia in the first day after surgery, suggested a longer hospitalization and additional therapy after discharge [22]. Furthermore, the incidence of SxH remained significantly different between groups (<70% vs >70%), even though excluded the patients with low iPTH (<1.6 pmol/L and <1 pmol/L). Recently published retrospective result, Raffaelli et al. also confirmed toxic goiter, and 25OH-VD deficiency are not risk factors for post-TT hypocalcemia. Relative parathyroid insufficiency seems to be the principal mechanism of postthyroidectomy hypocalcemia, even in patients with normal postoperative PTH concentrations [23]. Seo et al. proved that the absolute postoperative values and the relative decline >70% in PTH values in 1 h after surgery was reliable predictive factors for hypocalcemia [24]. A consensus was nearly reached that it would be more reasonable to take the baseline function of parathyroid into consideration, rather than the absolute number.

The retrospective nature of the present study is major inevitable weakness. Though the baseline is comparative in the study, a little part of patients underwent different extent of dissection, like bilateral and unilateral CND. This bias will be avoided in the conducting prospective study, which just include post-TT + BCND patients. Additionally, there lacks an illustration of continuing change of magnesium, calcium and iPTH, blood only was collected in the 1st morning after surgery. We believe it would be interesting to observe the change of electrolyte and iPTH in days after surgery. And we will investigate whether correction of hypomagnesemia could alleviate hypocalcemia, including symptoms in further prospective studies. Finally, we did not take the impact of proton pump inhibitor (PPI) on hypocalcemia and hypomagnesemia post-thyroidectomy. Several cases and researches found the potential link between chronic use of PPI and hypomagnesemia in small proportion of patients, though mechanism is not well-recognized [25, 26].

On the other hand, the strength of the present study is also obvious. A prospective database about thyroidectomy patients is maintained, though it is a retrospective study. It records the symptoms and biochemical data prospectively. Besides, all of less than toal thyroidectomy is excluded from the final analysis. And all of operations were performed by one surgeon. Additionally, data integrity is well in our study, every patient had perioperative data about electrolyte and iPTH.

## Conclusions

Hypomagnesemia is not a rare complication successive to thyroidectomy. 23.36% of patients developed hypomagnesemia, which proved to be independent risk factor for biochemical hypocalcemia in the study, other than the variable sex. And relative decline of iPTH, outweighed absolute value of iPTH and magnesium, was identified to be risk factor of symptomatic hypocalcemia. The result still remained significant when excluded hypoparthyroidism patients.

## Abbreviations

CND: Central nodal dissection
CT: Completion thyroidectomy
Po: Postoperative
PTG: Parathyroid gland
SxH: Symptomatic hypocalcemia
TTx: Total thyroidectomy
